# P19 Evaluation of vancomycin prescribing practice in MRSA-colonized neutropenic sepsis patients in St Bartholomew’s Hospital, London

**DOI:** 10.1093/jacamr/dlaf118.026

**Published:** 2025-07-14

**Authors:** Charlotte David, Kate David, Kenrick Ng

**Affiliations:** Barts Health NHS Trust, London, UK; Barts Health NHS Trust, London, UK; Barts Health NHS Trust, London, UK

## Abstract

**Background:**

Patients with cancer are susceptible to bacteraemias by virtue of their recurrent admissions, invasive procedures, immunosuppression and burden of disease. They are frequently exposed to broad-spectrum antibiotics and prolonged admissions, increasing their risk of colonization with resistant organisms such as MRSA) The outcome of MRSA bacteraemias are significantly worse than *Staphylococcus aureus* bacteraemias. Trust guidelines recommend empirical vancomycin for haematology/oncology patients with neutropenic sepsis with an MRSA-positive screen within the last 12 months.

**Objectives:**

Reduce MRSA-related sepsis in haematology/oncology patients, ensuring safe vancomycin prescribing to reduce resistance and toxicity.

**Methods:**

Barts Cancer Centre is the second largest cancer centre in London. Data for episodes of neutropenic sepsis in MRSA-positive haematology/oncology patients, January 2022 - December 2024, St Bartholomew’s Hospital, were extracted from electronic patient records. Episodes were excluded if the MRSA-positive screen was more than 12 months prior. Data included patient demographics, admission details, presence and nature of vancomycin prescription (empirical/delayed, indication) alongside relevant microbiology. Data were analysed as proportions or percentages.

**Results:**

31 episodes of neutropenic sepsis in 13 patients (46% female, 54% male) were identified. In 54.8% no source was identified, otherwise respiratory infection was the leading source of sepsis (12.9%), followed by soft tissue infections (9.7%).There were 7 Gram-positive blood cultures; 57.1% coagulase-negative staphylococci, 28.6% MRSA and 14.3% *Enterococcus faecium*. Empirical vancomycin was commenced in two cases due to severe penicillin allergy and known recent MRSA bacteraemia. Delayed vancomycin prescriptions occurred in 5 episodes due to Microbiology advice (80%) or Gram-positive bacteraemia (20%). The mean duration of vancomycin prescription was 4.8 days. Vancomycin was always discontinued appropriately. There were two episodes of MRSA bacteraemia in one patient during the study period; the first secondary to skin/soft tissue infection and no source identified in the second. This equates to a 5% annual incidence rate of MRSA bacteraemia in this patient cohort.

**Conclusions:**

Trust guideline adherence rate was 6.5%. Despite the mortality benefit of vancomycin, it confers a risk of nephrotoxicity and the morbidity and financial sequelae of this. Vancomycin-resistant enterococci (VRE) colonization may also arise, leading to complex, difficult to treat infections requiring toxic antimicrobials, increased mortality and contributing to global antimicrobial resistance. Given the relative infrequency of MRSA bacteraemia and the associated risk of vancomycin, we updated the Trust guideline to a more nuanced approach; to consider empirical vancomycin if there is a reasonable indication (e.g. previous MRSA bacteraemia). Otherwise, a delayed prescription can be adopted upon knowledge of up-to-date microbiology or in clinical deterioration. There is a fine balance between safeguarding for rare but grave outcomes whilst limiting adverse effects from toxic drugs. We believe that supporting the clinician’s assessment, backed by evidence and advice, will balance the risks and benefits as best as possible. Findings were presented locally and a further cycle will be conducted.

